# Journal of Anesthesia (JA) clinical reports

**DOI:** 10.1186/s40981-015-0010-9

**Published:** 2015-08-27

**Authors:** Michiaki Yamakage

**Affiliations:** Department of Anesthesiology, Sapporo Medical University School of Medicine, S1W16, Chuo-ku, Sapporo, Hokkaido 060-8543 Japan

Celebrating our inaugural edition!

We are delighted to launch our first issue of the Journal of Anesthesia (JA) Clinical Reports. Since manuscript submission began on April 22, we have already received over 20 papers, the first seven of which to be accepted comprise this issue. The journal’s aim is to offer a publication in which members of the Japanese Society of Anesthesiologists (JSA) can share their experiences with everyday cases and simple clinical studies with readers, thus helping to raise the standard of clinical anesthesiology. Of course, the overall purpose is so that patients can enjoy the benefits, but a further goal is to encourage JSA members, particularly young anesthesiologists, to submit papers in English. Although JA avoids publishing large numbers of case reports with low citation rates in order to improve its impact factor, this does not diminish the value of such reports.

The first paper published in this inaugural edition addresses methods of anesthesia used for Cesarean sections. Suzuki et al*.* carried out a study of anesthesia methods in patients who underwent Cesarean section in their hospital. Despite the study’s limitations due to the retrospective study, they suggest that the use of morphine in combination with spinal anesthesia may be useful in terms of early ambulation and postoperative pain relief compared with combined spinal and epidural anesthesia (CSEA) without the use of opioids. CSEA is the main method used in my own hospital, and this paper may spark reconsideration of anesthesia methods. Kusaka et al*.* report a case of persistent left superior vena cava. This in itself is a comparatively common congenital deformity, but cases in which the right superior vena cava is missing are extremely rare. Normally, patients do not display any symptoms and have no difficulty leading a normal life, but if they require some sort of treatment, there may be issues for us as anesthesiologists during procedures such as placing of a central venous catheter or pacing lead; therefore, this is a condition of which we should be aware. Other articles cover the successful management of a case of disseminated intravascular coagulation (DIC) due to amniotic fluid embolism, a case of postoperative acute transverse myelitis, airway management in a case of ossification of the anterior longitudinal ligament of the cervical spine, a case of successful control of systolic anterior motion (SAM) following mitral valve repair, and a case of interference in pulse oximetry by an optical navigation system used in CT-guided stereotaxic neurosurgery. By reading these cases, anesthesiologists will react differently: one might say “I hadn’t heard about that method of management before,” while another might think “In their shoes, I would have done things slightly differently.” All are case reports that will offer valuable insights for any readers.

In addition to publishing a journal that will be clinically useful, we look forward to receiving a constant stream of submissions by JSA members describing their experience in treating important clinical cases. We also want to challenge young anesthesiologists to write up and submit their interesting cases in English, and hope that senior doctors will give them this opportunity.

More in our next issue!

